# Expression profile analysis of LncRNAs and mRNAs in pre-receptive endometrium of women with polycystic ovary syndrome undergoing in vitro fertilization-embryo transfer

**DOI:** 10.1186/s12920-024-01806-w

**Published:** 2024-01-19

**Authors:** Xiuhua Xu, Aimin Yang, Pengxiang Tian, Kun Zhang, Yuanyuan Liu, Yizhuo Wang, Ziwei Wang, Yanjing Wu, Zhiming Zhao, Qian Li, Baojun shi, Xianghua Huang, Gui-min Hao

**Affiliations:** 1 Hebei Key Laboratory of Infertility and Genetics, Hebei Clinical Research Center for Birth Defects, Hebei Medical Key Discipline of Reproductive Medicine, Hebei Collaborative Innovation Center of Integrated Traditional and Western Medicine on Reproductive Disease, Department of Reproductive Medicine, Second Hospital of Hebei Medical University, Shijiazhuang, 050000 China; 2 Department of Gynecology and Obstetrics, Second Hospital of Hebei Medical University, Shijiazhuang, 050000 China; 3 Cardiovascular platform, Institute of Health and Disease, Hebei Medical University, Shijiazhuang, 050000 China

**Keywords:** PCOS, Pre-receptive endometrium, Endometrial receptivity, lncRNA, lncRNA-mRNA network

## Abstract

**Background:**

To compare the expression levels of long non-coding RNA (lncRNA) and messenger RNA (mRNA) in pre-receptive endometrium between patients with Polycystic Ovary Syndrome (PCOS)and normal ovulation undergoing in vitro fertilization-embryo transfer (IVF-ET).

**Methods:**

Endometrial tissues were collected with endometrial vacuum curette in pre-receptive phase (3 days after oocytes retrieval) from PCOS and control groups. LncRNAs and mRNAs of endometrium were identified via RNA sequencing and alignments. A subset of 9 differentially expressed lncRNAs and 11 mRNAs were validated by quantitative reverse transcription polymerase chain reaction(qRT-PCR)in 22 PCOS patients and 18 ovulation patients. The function of mRNAs with differential expression patterns were explored using Gene Ontology (GO) and Kyoto Encyclopedia of Genes and Genomes (KEGG).

**Results:**

We found out 687 up-regulated and 680 down-regulated mRNAs, as well as 345 up-regulated and 63 down-regulated lncRNAs in the PCOS patients in contrast to normal ovulation patients. qRT-PCR was used to detect the expression of 11 mRNAs, and validated that the expression of these 6 mRNAs CXCR4, RABL6, OPN3, SYBU, IDH1, NOP10 were significantly elevated among PCOS patients, and the expression of ZEB1 was significantly decreased. qRT-PCR was performed to detect the expression of 9 lncRNAs, and validated that the expression of these 7 lncRNAs IDH1-AS1, PCAT14, FTX, DANCR, PRKCQ-AS1, SNHG8, TPT1-AS1 were significantly enhanced among PCOS patients. Bioinformatics analysis showed that differentially expressed genes (DEGs) involved KEGG pathway were tyrosine metabolism, PI3K-Akt pathway, metabolic pathway, Jak-STAT pathway, pyruvate metabolism, protein processing in endoplasmic reticulum, oxidative phosphorylation and proteasome. The up-regulation of GO classification was involved in ATP metabolic process, oxidative phosphorylation, RNA catabolic process, and down-regulation of GO classification was response to corticosteroid, steroid hormone, and T cell activation.

**Conclusion:**

Our results determined the characteristics and expression profile of endometrial lncRNAs and mRNAs in PCOS patients in pre-receptive phase, which is the day 3 after oocytes retrival. The possible pathways and related genes of endometrial receptivity disorders were found, and those lncRNAs may be developed as a predictive biomarker of endometrium in pre-receptive phase.

## Background

Polycystic ovary syndrome (PCOS) is a common ovulation disorder in women of reproductive age. This syndrome causes infertility, insulin resistance, obesity, endometrial hyperplasia, and cardiovascular problems. There are differences in endometrium between PCOS women and healthy controls [1]. Endometrial components are responsible for low fertility and poor pregnancy outcomes in women.

A number of clinical and biochemical factors associated with PCOS could potentially undermine the function of endometrium during pre-receptive, receptive and post-receptive phases [1–3]. From a number of interventions to improve endometrial receptivity, there are substantial evidence that endometrial dysfunction increases the risk of pregnancy complications in women with PCOS [4]. It is speculated that the clinical characteristics of the PCOS patients may contribute to dysregulated sex hormone receptor expression and insulin resistance, as well as chronic inflammation, and abnormal vascularity and immune function in the endometrium. Endometrial dysfunction, abnormal trophoblast infiltration, and placental formation in women with PCOS can lead to miscarriage and pregnancy complications. The mechanisms of endometrial dysfunction in patients with PCOS are not well understood.

Long non-coding RNAs (lncRNAs) are a diverse class of RNAs which are more than 200 nucleotides in length and play important role in numerous biological processes. There are some preliminary studies on the role of lncRNAs in PCOS. It was reported that the expression of lncRNA ENST00000550337.1 in PCOS patients was significantly higher than the control group [5]. The lncRNA ENST00000550337.1 in peripheral blood is mainly participate in glucose metabolism. In a Chinese preliminary study, long non-coding RNA H19 was linked to PCOS [6]. In addition, Zhang et al. found that lncRNA CD36-005 may regulated mRNAs related to endometrium function in endometrial stromal cells of PCOS rat model [7]. LncRNA CD36-005 was significantly up-regulated in the ovaries and uterus of PCOS rat model. Moreover, lncRNA CD36-005 overexpression could change the expression of mRNAs in endometrial stromal cells of rat PCOS model, which participate in many biological processes [7]. However, there are few studies focused on the function of lncRNA in the pathogenesis of endometrial in PCOS patients. Hundreds of differentially expressed genes (DEG)s have been identified in the endometrium of PCOS patients during WOI [8–11]. Endometrium is a dynamic tissue; it undergoes cyclic changes under the control of steroid hormone. Studies reported on the transcriptomic profiling of endometrium across the menstrual cycle of natural cycle [12, 13] and COS cycles [14–16]. According to the receptivity of the endometrium to the embryo, the secretory phase can be divided into pre-receptive, receptive, and post-receptive endometrium. However, there have been no reports of DEGs in pre-receptive endometrium of PCOS patients after receiving COS. Therefore, this study was to investigate the different expression levels of lncRNA and mRNA in pre-receptive endometrium between PCOS patients and normal ovulation undergoing IVF-ET.

## Materials and methods

### Ethics statement

PCOS patients and patients with normal ovulation underwent IVF/ICSI-ET at our department were enrolled from October 2019 to March 2021. This study was approved by the institutional review board of the Second Hospital of Hebei Medical University (Ethics No. 2018-R028).

### IVF-ET protocol and patient recruitment

40 patients (22 PCOS patients and 18 patients with normal ovulation) underwent IVF/ICSI-ET were enrolled. PCOS was diagnosed by the Rotterdam consensus. Flexible GnRHant protocol was applied for all the included patients. COS was started from day 2–3 of menstrual bleeding or withdrawal bleeding. Recombinant FSH (r-FSH; GonalF, Switzerland; Merck, Serono, China; Pouliquen, Merck Sharp & Dohme, China; Vermont, IBSA, China) and/or human menopausal gonadotropin (HMG; Livzon, China, or Menopur, Hui Ling, China) were used for COS. GnRHant 0.25 mg was used when the leading follicle reached 14 mm or serum estrogen level exceed 200ng/mL [17]. When ≥ 3 dominant follicles reached 17 mm in diameter, 250ug recombinant hCG (r-hCG) (r-HCG; Ovitrelle, Switzerland; Merck Serono) was used for trigger. Oocyte retrieval was performed 35.5-36.5 h after hCG administration. Patients who did not receive fresh embryo transfer due to mild or moderate OHSS were included in our study. In addition, patients with the progesterone level exceed 1.5ng/ml or the endometrium thickness under 7 mm in the trigger day was excluded. From the morning of the oocyte pickup day, 90 mg progesterone gel (Crinone, Merck Serono, Watford, UK) was administered.

### Endometrium collection

Endometrium was collected in 3 patients in both groups on day 3 after oocyte retrieval. Each endometrial tissue sample was randomly selected from PCOS group and normal ovulation group for RNA extraction. The patient assumed the lithotomy position on the operating table after emptying the bladder. We used one-off endometrium suction tube to aspirate endometrial tissue. In order to prevent infection, antibiotics were given for 3 days after the surgery. Bathing and intercourse were forbidden for 2 weeks.

### RNA isolation and RNA-seq

Total RNA was extracted from 100 mg of endometrial samples by homogenization with 1 ml of TRIzol Reagent (Invitrogen, Carlsbad, CA). The concentration was determined using the Qubit RNA assay kit in the Qubit 2.0 Flurometer (Life Technologies, Carlsbad, CA, USA). The RNA integrity was assessed using Agilent Bioanalyzer 2100 (Agilent Technologies). All RNA samples were stored at -80 °C for further analysis when the ratio of absorbance at 260 and 280 nm was between 1.8 and 2.2.

After removing ribosomal RNA, nucleotides were used to build a chain specific library [18]. Firstly, the RNA was fragmented into 250-300 bp fragments. Single-strand cDNA sythesis was conducted using fragmented RNA templates and random oligonucleotide primers. After that, the RNA templates were removed by RNase H-mediated degradation. Then complementary DNA strand was made using dNTP substrates, forming double-stranded DNA after adenylation of the 3’ ends and purification. cDNA of about 200 bp were screened using AMPure XP beads. After degradation of strands containing U with USER enzyme, PCR amplification was performed to obtain the library. A final assessment of library quality was conducted using the Agilent Bioanalyzer 2100, and NDEX-coded samples were clustered using the TruSeq PE Cluster Kit v3-cBot-HS (Illumia). Illumina Hiseq 4000 was used to sequence the clustered libraries and generate paired-end reads of 150 bp. The Novogene Co. LTD, Beijing, China performed all the RNA-Seq and data collection.

### RNA-seq data and enrichment analysis

The genome comparison of clean reads to the genome or transcriptome is the basis of subsequent analysis. The RNA-Seq sequencing data were analyzed by Hisat2 software. We use Cuffmerge software to merge the transcripts obtained from sample stitching, remove the transcripts whose chain direction is uncertain and the transcript length is less than 200nt. Next, we use Cuffcompare software to compare with known databases and filter out known transcripts from databases. Finally, the coding potential of the selected transcripts was predicted, and Novel_lncRNA and Novel_mRNA were obtained. By referring to HGNC, the newly screened lncRNAs are divided into four types: antisense, lincRNA, sense overlapping, and sense intronic, based on the position relationship of known mRNAs. The network flow algorithm (StringTie) was applied to splice and quantify the transcripts and genes [19]. Cuffdiff or edgeR software was used for the differential gene expression. Padj ≤ 0.05 was selected as the significantly differentially expressed. Through the significant enrichment of pathway, we can explore the main biochemical metabolic pathways and signal transduction pathways involved in differentially expressed genes. We adopt clusterProfiler (http://www.bioconductor.org/packages/release/bioc/html/clusterProfiler.html) and used widely annotated gene databases GO and KEGG to analysis the pathway enrichment of differential genes. GO (Gene Ontology) is a comprehensive database describing gene functions, which can be categorized into molecular function, biological process and cellular component. KEGG (Kyoto Encyclopedia of Genes and Genomes) is a comprehensive database integrating genomic, chemical and system function information [20].

### Construction of lncRNA/mRNA interaction network

We constructed lncRNA-mRNA network using Cytoscape3.6.1 to investigate their interactions. Different shapes represent different RNA types. Pearson correlation coefficient was calculated with a significance threshold of *R* ≥ 0.95 or *R* ≤ − 0.95 to evaluate the relationship between lncRNAs and mRNAs. Significantly correlated pairs of lncRNA-mRNA were selected to draw the network.

### qRT-PCR

To verify the sequencing results, 22 PCOS patients and 18 ovulating patients were selected, and the expressions of 9 lncRNAs and 11 mRNAs were verified by qRT-PCR. After total RNA was extracted, cDNA was generated by reverse transcription. cDNA synthesis of lncRNA and mRNA according to the vendor’s instructions of the MonAmp™ Reverse Transcription Kit. The expression level of lncRNA and mRNA was evaluated by qRT-PCR using MonAmp™ ChemoHS qPCR Mix in a 10 µl reaction system, including 5 µl MonAmp™ ChemoHS qPCR Mix, 1 µl cDNA, 0.5 µl forward/reverse primers (10µmol/L), and 3.5 µl triple distilled water. The specific quantitative primers were designed using oligo7 software and synthesized by Sangon Biotech (Shanghai, China, Table 1). The cycling program was pre-denaturation: 95 °C, 10 min, denaturation: 95 °C, 10s, annealing and extension: 60 °C, 30s, repeat for 40 cycles. After 2^−ΔΔCT^ analysis, the expression levels were normalized to β-actin levels. Each sample had three individual technical replicates.

**Table 1:** Primer sequences for qRT-PCR.

**lncRNA**	**Forward primer (5’-3’)**	**Reverse primer (5’-3’)**
IDH1-AS1	TCAGACTCACAACCACAGCC	GACAAAGCCGGGAAGAGGAA
PCAT14	TGACCTTGTGATCTGCCCAC	ACGATTGTCTCCGTTCCTGG
FTX	TGAACAAAGCGGGGCAAATG	TGTAAAGCTCAGGGCCCTTG
DANCR	AGTTCTTAGCGCAGGTTGAC	AAGGTGAACATGAAGCACCT
PRKCQ-AS1	ACTGCTTTCAACTTTACTG	AGTCCTCAGCATTATTCC
SNHG8	CCCGAGAACCGTCAGTTTGA	ACACCCGTTTCCCCAACTAC
TPT1-AS1	GGTCAGCTCCAAGGAGGCTAT	GCCAGTGCTCTGAAGGAAAAC
TRPM2-AS	CGTGACCAGGTTCAGACACA	TGGGCAGTTTGGTTCTGGTT
DCST1-AS1	CCACTCACCAGCTTCTTC	CTTCTGCTATGTCTCACCC
**mRNA**	**Forward primer (5’-3’)**	**Reverse primer (5’-3’)**
CXCR4	ACTACACCGAGGAAATGGGCT	CCCACAATGCCAGTTAAGAAGA
RABL6	TGATCCGGGGAGACAGGAAC	CGATGTCATCCGTGGTCTTGTA
OPN3	CTGGTGCTCGTCCTCTACTAC	GGACACGAAGGTAAAGGTGAC
SYBU	TTCTTCACGCAATCGAGGTCC	GGGCTACAGTCGCTTCCTTT
IDH1	TGTGGTAGAGATGCAAGGAGA	TTGGTGACTTGGTCGTTGGTG
NOP10	CAGTATTACCTCAACGAGCAGG	GGCTGAGCAGGTCTGTTGTC
ZEB1	GATGATGAATGCGAGTCAGATGC	ACAGCAGTGTCTTGTTGTTGT
DKK1	CCTTGAACTCGGTTCTCAATTCC	CAATGGTCTGGTACTTATTCCCG
ANXA1	CTAAGCGAAACAATGCACAGC	CCTCCTCAAGGTGACCTGTAA
STC1	CACGAGCTGACTTCAACAGGA	GGATGTGCGTTTGATGTGGG
CST4	CCTCTGTGTACCCTGCTACTC	CTTCGGTGGCCTTGTTGTACT
β-actin	CATGTACGTTGCTATCCAGGC	CTCCTTAATGTCACGCACGAT

### Statistical analysis

Clinical analyses were calculated by SPSS 20.0 (IBM Corp, USA). Results were expressed as mean ± SD. Statistical comparison between the PCOS and the control group was performed by t-test for normal distribution. Unpaired-test and the Mann-Whitney U test were conducted for continuous variables. Categorical variables were used by chi-squared tests or Fisher’s exact tests. *P* < 0.05 was considered statistically significant.

## Results

### Clinical characteristics of participants

The clinical characteristics of patients were detailed in Table 2. There were no difference in age and BMI between PCOS patients and ovulation patients. The level of FSH, E2 and T had no difference between the two groups. PCOS patients had higher basal LH. There were no significant differences in HOMA-IR, occytes retrival and thickness of endometrium between the two groups.

**Table 2:** The baseline characteristics of participants

Variables	PCOS	OVULATE	*P*
Age(years)	29.00(5.5)	27.00(2.25)	0.559
BMI(kg/m^2^)	22.27 ± 3.24	23.26 ± 3.27	0.547
FSH(mIU/ml)	6.53 ± 1.51	6.19 ± 2.05	0.729
LH(mIU/ml)	9.42 ± 4.78	39.42 ± 21.32	0.004*
E_2_(pg/ml)	42.83 ± 18.84	39.41 ± 21.32	0.744
T(ng/ml)	0.689 ± 0.17	0.55 ± 0.24	0.247
HOMA-IR	3.10 ± 0.83	2.64 ± 0.68	0.455
Occytes retrival	22.5(2.5)	17(8)	0.517
Thickness of endometrium(mm)	11.00 ± 1.41	12.00 ± 1.82	0.46

PCOS: polycystic ovary syndrome, BMI: body mass index, FSH: follicle-stimulating hormone. LH: luteinizing hormone, E2: estradiol, T: testosterone, HOMA-IR: homeostasis model assessment of insulin resistance. Continuous data was presented as mean ± SD (normally distributed) and median (25th percentile-75th percentile) (non-normally distributed)

*P* < 0.05

### Differentially expression profiles of lncRNAs and mRNAs in pre-receptive endometrium of PCOS and ovulation groups

**Fig. 1:**
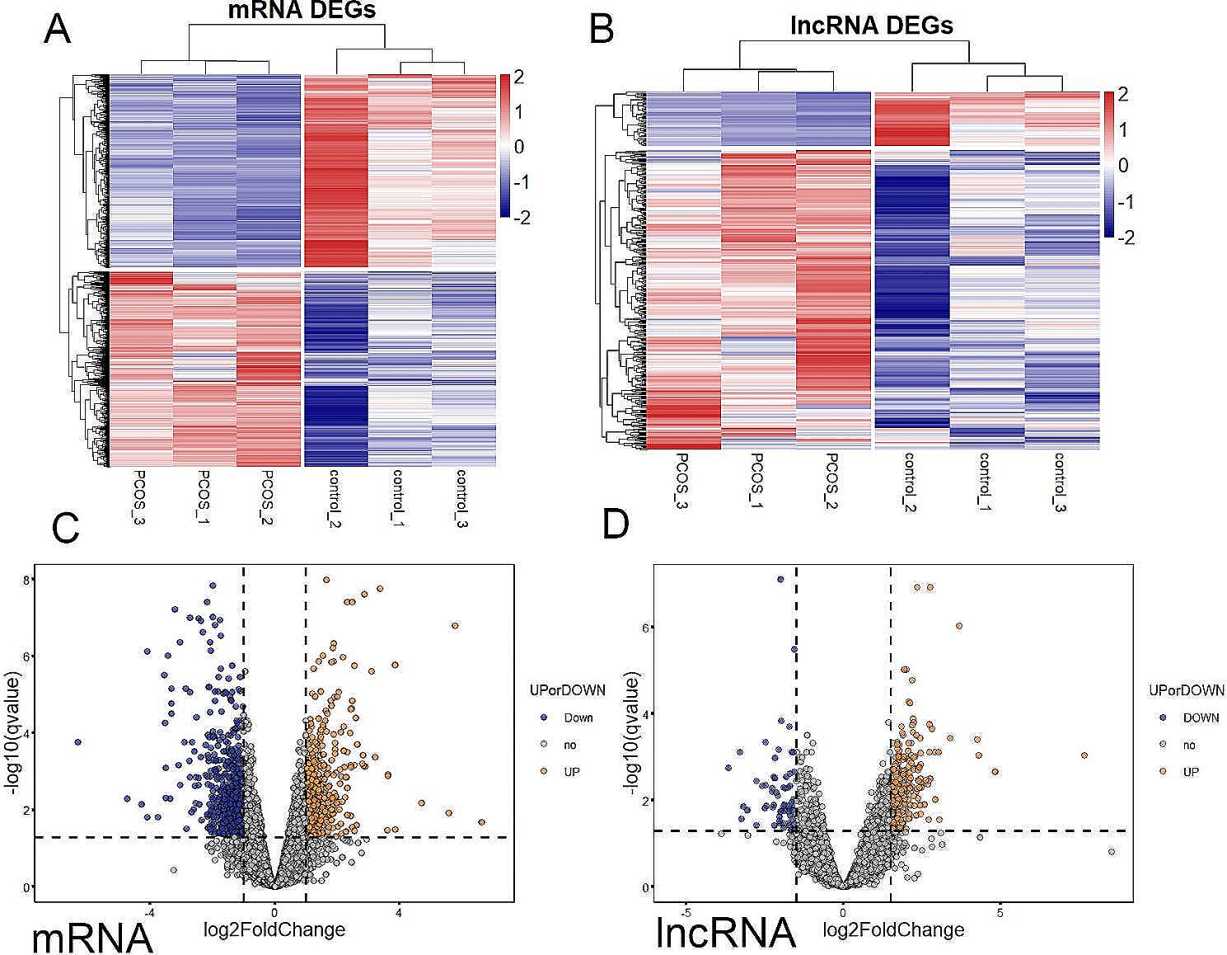
lncRNA and mRNA expression profile of endometrium between PCOS and control samples. (A) Hierarchical cluster heat map of mRNA expression in PCOS patients and controls. (B) Hierarchical cluster heat map of lncRNA expression in PCOS patients and controls. Red and blue represent up- and down- regulated genes respectively. (C) Volcano plot of differentially expressed mRNAs between PCOS patients and controls. (D) Volcano plot of differentially expressed lncRNAs between PCOS patients and controls. The orange and blue plots represent the statistically significantly up- and down-expressed mRNA and lncRNA respectively

To investigate the expression profiles of lncRNAs and mRNAs in endometrium with PCOS patients, 6 women (3 with PCOS and 3 matched ovulation patients) were recruited and endometrium was collected on 3 days after oocytes retrival for RNA-seq analysis. RNA-seq results showed that the expression patterns of lncRNAs and mRNAs in PCOS patients were different from those in the controls. Differentially expressed genes (DEGs) of PCOS were calculated using the data of GSE26787, and then re-annotated as differentially expressed mRNAs (DEMs) and lncRNAs (DELs).Topological analysis determined the key mRNAs and lncRNAs with the highest centroid. There were 687 up-regulated mRNAs and 680 down-regulated mRNAs (log_2_FC ≥ 1.0 and *P*adj ≤ 0.05), 345 up-regulated lncRNAs and 63 down-regulated lncRNAs (log_2_FC ≥ 1.5 and *P*adj ≤ 0.05) in the PCOS patients. Hierarchical clustering analysis showed the overview of mRNAs and lncRNAs expression (Fig. 1A,B). The volcano plots displayed the variation in mRNAs and lncRNAs expression between PCOS and Control groups (Fig. 1C,D). The names of the top 20 up-regulated and 20 down-regulated mRNAs and lncRNAs with differential expressed were shown in Tables 3 and 4 respectively. ANO3 was the most significantly up-regulated mRNA with a log_2_FC of 6.65. The expression of PAEP mRNA was down-regulated most significantly, with a log_2_FC of -6.31. For lncRNAs, the most up-regulated lncRNAs was AC122108.1 with a log_2_FC of 7.66. The most down-regulated lncRNAs was AC104072.1 with a log_2_FC of -3.66.

**Table 3:** Top 20 up-regulated and down-regulated mRNAs in PCOS compared with control

mRNA	baseMean	log_2_FoldChange	lfcSE	stat	*p*value	padj
**The top 20 up-regulated mRNAs**						
ANO3	157.3071602	6.648539703	2.221707526	2.992535977	0.002766701	0.02094944
ZCCHC12	122.5225693	5.784659671	0.915759628	6.316788262	2.67E-10	1.57E-07
CALCR	2421.815726	5.596638064	1.719322106	3.255142271	0.001133356	0.011796194
CADM2	40.41806472	4.723687436	1.337086642	3.532820752	0.000411151	0.006439965
AC005833.1	171.5341571	3.932053086	0.53552034	7.342490637	2.10E-13	4.01E-10
PCSK5	9402.966711	3.87014543	1.39044394	2.783388326	0.005379438	0.03224502
CST4	60.51995148	3.858555554	0.658165445	5.862592125	4.56E-09	1.70E-06
KIF1A	113.1998088	3.641537817	0.87558267	4.158987998	3.20E-05	0.001287307
DISP3	39.22201926	3.635825654	0.867074529	4.193210076	2.75E-05	0.001165887
SPDEF	1512.491113	3.606229488	1.305902049	2.761485435	0.005753908	0.033483351
MMEL1	64.13007612	3.376385323	0.500995444	6.739353354	1.59E-11	1.74E-08
KMO	2684.853597	3.283212864	0.434940609	7.548646405	4.40E-14	1.12E-10
DNAH8	140.5014962	3.216801926	0.708432865	4.540729386	5.61E-06	0.000420532
ADCYAP1R1	1656.535652	3.108806719	0.537929504	5.779208418	7.51E-09	2.50E-06
ELFN2	33.59811717	2.912199046	0.669790149	4.347927555	1.37E-05	0.000732784
TNFSF11	97.99024493	2.871329047	0.429686644	6.682379101	2.35E-11	2.40E-08
GABRQ	69.81371357	2.860582962	0.655315798	4.365197618	1.27E-05	0.000696631
FAM189A1	32.33711087	2.840343239	0.620961448	4.574105601	4.78E-06	0.000373408
MAT1A	80.78973322	2.821342751	0.639015503	4.415139753	1.01E-05	0.000612999
PRKCQ	1278.233547	2.813881386	0.386510519	7.280219416	3.33E-13	5.67E-10
**The top 20 down-regulated mRNAs**						
PAEP	290.1337998	-6.312976975	1.318491044	-4.788031747	1.68E-06	0.000174149
STC1	1972.76416	-4.746556644	1.30464222	-3.638205611	0.000274544	0.005098726
CXCL14	400.7516327	-4.291213538	1.227244682	-3.496624267	0.000471185	0.007059697
SLC15A1	481.9399755	-4.09932095	1.307254123	-3.135825605	0.001713711	0.015336208
SIK1	436.8752207	-4.097336539	0.679866407	-6.026678918	1.67E-09	7.53E-07
S100P	31.45381157	-3.763955105	1.203275811	-3.128090061	0.001759463	0.015599685
GABRE	322.7174059	-3.544738283	0.617756874	-5.738079871	9.58E-09	3.05E-06
AOX1	340.7856146	-3.530563746	0.694030375	-5.087044996	3.64E-07	5.46E-05
KCND2	199.0849394	-3.507598611	0.810875439	-4.325693493	1.52E-05	0.000785373
RIMKLB	7607.02118	-3.502822337	0.957460025	-3.65845283	0.000253742	0.004833396
GABRA2	548.6255526	-3.432582493	0.574065464	-5.979426927	2.24E-09	9.64E-07
ATP12A	39.57450851	-3.355914733	0.923047861	-3.63568876	0.000277239	0.005123897
ATF3	334.1369043	-3.328213755	0.5971486	-5.573510103	2.50E-08	6.95E-06
THBS2	700.6283886	-3.327947103	0.637604448	-5.219454023	1.79E-07	3.13E-05
C1orf116	33.82118676	-3.315628628	0.619043237	-5.35605339	8.51E-08	1.67E-05
OR2A4	439.643488	-3.245233566	0.329932394	-9.836056174	7.87E-23	1.20E-18
NR4A1	698.0154014	-3.210500308	0.492890899	-6.513612476	7.34E-11	5.91E-08
C2CD4A	1225.298302	-3.107462632	0.388523924	-7.998124295	1.26E-15	4.83E-12
PRSS51	143.1514225	-3.084718579	0.703500999	-4.384810519	1.16E-05	0.000667562
LEFTY2	94.98716252	-3.049740569	0.769103193	-3.965320384	7.33E-05	0.002238863

baseMean: mean of normalized counts for all samples, log_2_FoldChange: log_2_ fold change (MLE), lfcSE: standard error, stat: Wald statistic, *p*value: Wald test *p*-value, padj: BH adjusted *p*-values

**Table 4:** Top 20 up-regulated and down-regulated lncRNAs in PCOS compared with control

lncRNA	baseMean	log_2_FoldChange	lfcSE	stat	*p*value	padj
**The top 20 up-regulated lncRNAs**						
AC122108.1	677.891629	7.660959142	1.681903464	4.554933921	5.2402E-06	0.000914135
FP236383.6	29.58834652	4.823486434	1.139638313	4.232471283	2.31137E-05	0.002192215
AL392023.1	29.46618757	4.303728522	0.943645176	4.560748711	5.09716E-06	0.000905347
AL139424.1	35.11602524	4.252076925	0.871281444	4.880256493	1.05948E-06	0.000383335
AC108861.1	125.2648473	3.676374861	0.587128775	6.261615875	3.81009E-10	9.30518E-07
LINC02648	63.64769614	3.385687304	0.690088615	4.906163109	9.28753E-07	0.000361507
AC084866.1	1407.65649	3.059716852	0.989344755	3.092670009	0.001983646	0.027762515
FP236383.4	49.55393869	3.021730453	0.655597028	4.609127747	4.04362E-06	0.00078215
FP236383.5	49.55393869	3.021730453	0.655597028	4.609127747	4.04362E-06	0.00078215
FP671120.6	49.55393869	3.021730453	0.655597028	4.609127747	4.04362E-06	0.00078215
AL590064.1	3089.858553	2.923794161	0.634336218	4.609218393	4.04186E-06	0.00078215
AL355994.3	29.2078309	2.923252937	0.818107872	3.573187641	0.000352662	0.009423619
LINC02128	434.9221285	2.824974129	0.559081211	5.052886903	4.35182E-07	0.000223752
AC104248.1	73.13669721	2.760898522	0.678191858	4.070969727	4.68178E-05	0.003094996
AL136084.1	305.7556547	2.75644737	0.413815637	6.661051738	2.71875E-11	1.18164E-07
DCST1-AS1	44.77792196	2.733179143	0.589659434	4.635182593	3.56623E-06	0.00078215
AC091544.6	91.67440241	2.724120342	0.529759855	5.142179639	2.71569E-07	0.000176864
AC133485.7	400.8456805	2.706635857	0.677232137	3.996614617	6.42548E-05	0.003490222
AC010624.5	39.12990377	2.698465547	0.873007841	3.090998064	0.001994849	0.02783873
AC010494.1	17.28591171	2.680011891	0.683865274	3.91891794	8.89474E-05	0.004259446
**The top 20 down-regulated lncRNAs**						
AC104072.1	15.92732625	-3.667692743	0.847795577	-4.32615225	1.52E-05	0.001806569
A2ML1-AS1	194.2296643	-3.284871646	0.707738334	-4.641364592	3.46E-06	0.00078215
AC012593.2	571.0705466	-3.248593121	1.03995641	-3.123778161	0.00178545	0.026268166
AP006248.2	20.5378044	-3.181916155	0.935456275	-3.40145899	0.000670272	0.013872639
AL596188.1	18.10031166	-3.072928351	0.926906633	-3.315251226	0.000915608	0.016564026
AL021368.1	18.91135996	-2.764218892	0.939878373	-2.941038939	0.003271134	0.037862215
AC104964.3	196.1774362	-2.760069478	0.690771	-3.995636005	6.45E-05	0.003490222
AC097504.2	25.52072952	-2.590827947	0.668056453	-3.878157203	0.000105251	0.00467361
AC023043.3	21.41383491	-2.48781474	0.737797834	-3.371946386	0.00074639	0.014711767
AP002478.1	170.8390561	-2.479054966	0.51220236	-4.839991303	1.30E-06	0.000453019
AC011891.2	20.37659315	-2.466548902	0.630992501	-3.908998757	9.27E-05	0.004358306
AC007877.1	22.83532487	-2.454226204	0.717432636	-3.4208455	0.000624268	0.013324489
AC012213.1	111.4005541	-2.280638187	0.597053029	-3.81982517	0.000133546	0.005197664
AC073218.1	124.9401135	-2.269555805	0.607480616	-3.736013538	0.000186961	0.006191248
AC096733.1	17.00283172	-2.246840872	0.663220647	-3.387772804	0.000704626	0.014192767
AC012404.2	82.00179592	-2.225090298	0.539853219	-4.121657928	3.76E-05	0.002762904
AC007448.3	25.6859932	-2.22038662	0.644395706	-3.445688108	0.000569607	0.012704325
LINC02432	805.228432	-2.203462113	0.540245203	-4.078633369	4.53E-05	0.003062781
AC139720.2	27.17049046	-2.191699338	0.744392355	-2.944279751	0.003237073	0.03778261
NR4A1AS	143.8088869	-2.174589693	0.532679191	-4.08236276	4.46E-05	0.003055715

### Prediction of gene phenotypes based on GO annotations and KEGG pathways

**Fig. 2:**
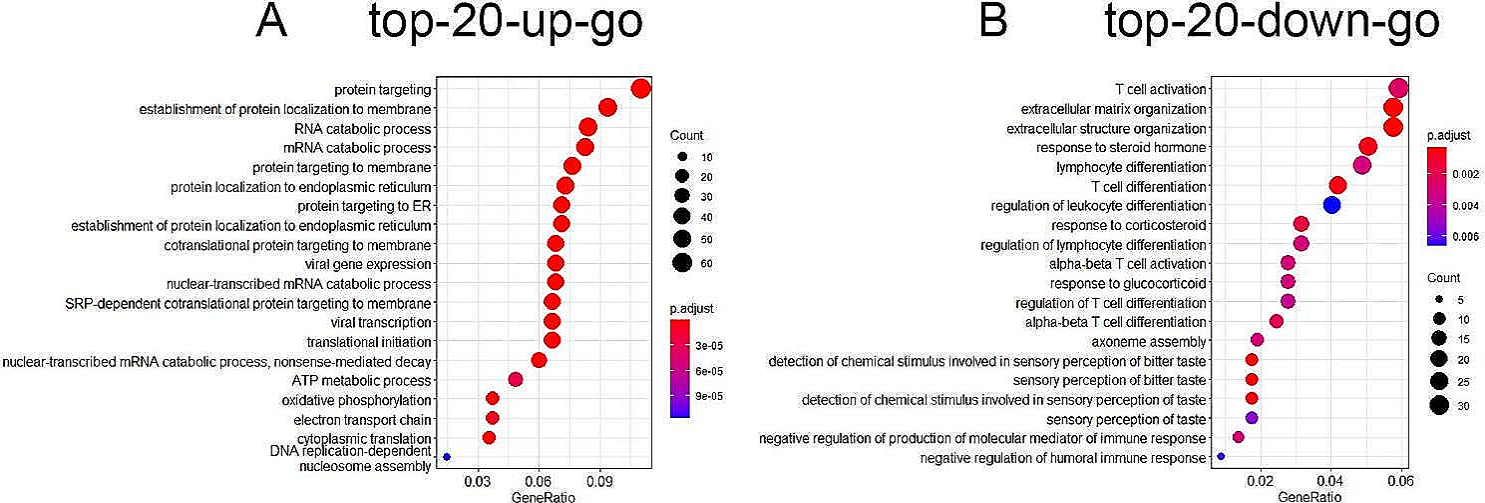
GO analysis of differentially expressed mRNAs in PCOS patients and controls. (A) The top 20 up GO terms for the different expressed mRNA in women with PCOS versus a control group. (B) The top 20 down GO terms for the different expressed mRNA in women with PCOS versus a control group. The left side represents the annotation terms

**Fig. 3:**
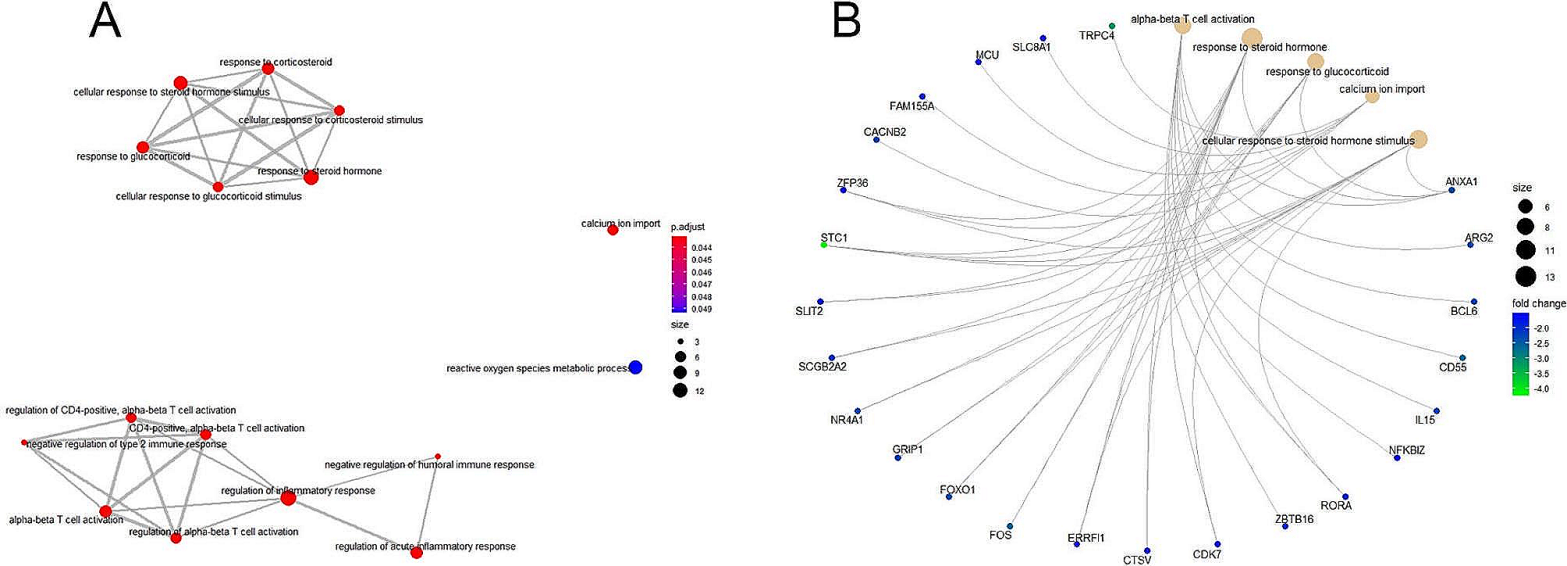
Weighted gene co-expression network analysis of differentially expressed genes associated with PCOS patients. (A) Corticosteroid, steroid hormone, and glucocorticoid related GO terms were enriched. T cell activation and regulation of inflammatory response related GO terms were enriched. (B) Common genes in GO terms for alpha-bata T cell activation, response to steroid hormone, response to glucocorticoid, calcium ion import and cellular response to steroid hormone stimulus were analyzed and enriched

We used GO and KEGG pathway enrichment analyses to investigate the potential functions of differentially expressed mRNAs. The top 20 up- and 20 down-GO annotations on biological processes were shown in Fig. 2A,B. The up-regulation of GO classification was involved in protein targeting to membrane, protein targeting to ER, establishment of protein localization to membrane, RNA catabolic process, protein localization to endoplasmic reticulum, ATP metabolic process and oxidative phosphorylation. The down-regulation of GO classification was T cell activation, extracellular matrix organization, extracellular structure organization, response to steroid hormone, T cell differentiation, regulation of leukocyte differentiation, response to corticosteroid, response to glucocorticoid, sensory perception of taste, negative regulation of humoral immune response. We found that response to corticosteroid, steroid hormone, and glucocorticoid related GO terms were enriched. The T cell activation and regulation of inflammatory response related GO terms were enriched (Fig. 3A). Common genes in GO terms for alpha-bata T cell activation, response to steroid hormone, response to glucocorticoid, calcium ion import and cellular response to steroid hormone stimulus were ZFP36, STC1, SLIT2, SCGB2A2, NR4A1,GRIP1, FOXO1, FOS, ERRFI1,CTSV,CDK7, RORA, ANXA1(Fig. 3B).

**Fig. 4:**
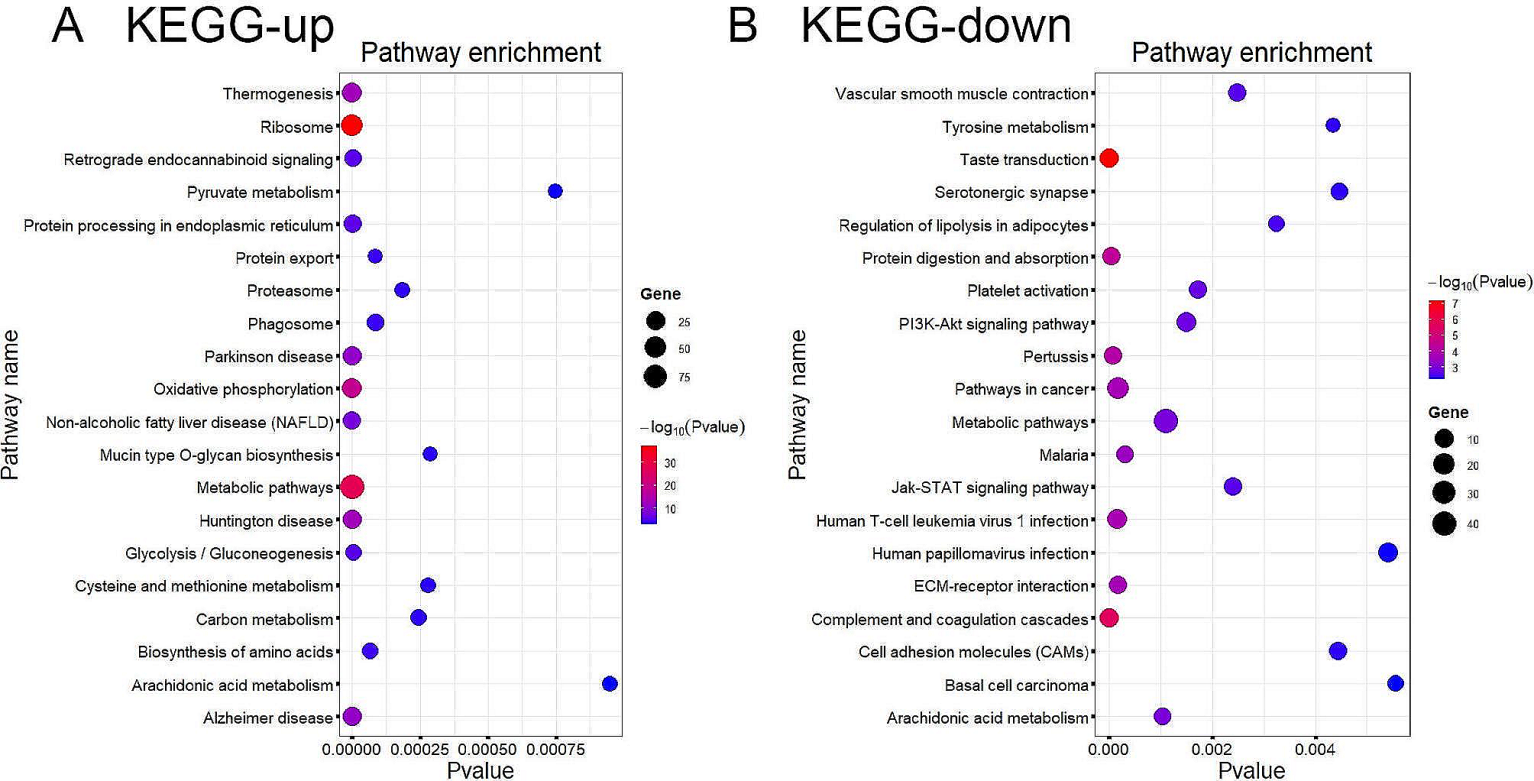
KEGG pathway analysis of differentially expressed mRNAs in PCOS patients and controls. (A) The top 20 up KEGG terms for the different expressed mRNA in women with PCOS versus a control group. (B) The top 20 down KEGG terms for the different expressed mRNA in women with PCOS versus a control group. The horizontal axis represents the p value, and the vertical axis represents the pathway name. The size of the dot indicates the number of differentially expressed genes in the pathway, and the color of the dot corresponds to the different–log10(p-value)

Bio-informatics analysis showed that DEGs involved up-regulated KEGG [20] pathway (Fig. 4A) were thermogenesis, ribosome, retrograde endocannabinoid signaling, pyruvate metabolism, protein processing in endoplasmic reticulum, oxidative phosphorylation and metabolic pathway. DEGs involved down-regulated KEGG [20] pathway (Fig. 4B) were vascular smooth muscle contraction, tyrosine metabolism, PI3K-Akt signaling pathway, metabolic pathway, Jak-STAT signaling pathway, ECM-receptor interaction, complement and coagulation cascades, cell adhesion molecules (CAMs).

### Analysis of lncRNA-mRNA co-expression profiles in pre-receptive endometrium of patients with PCOS

**Fig. 5:**
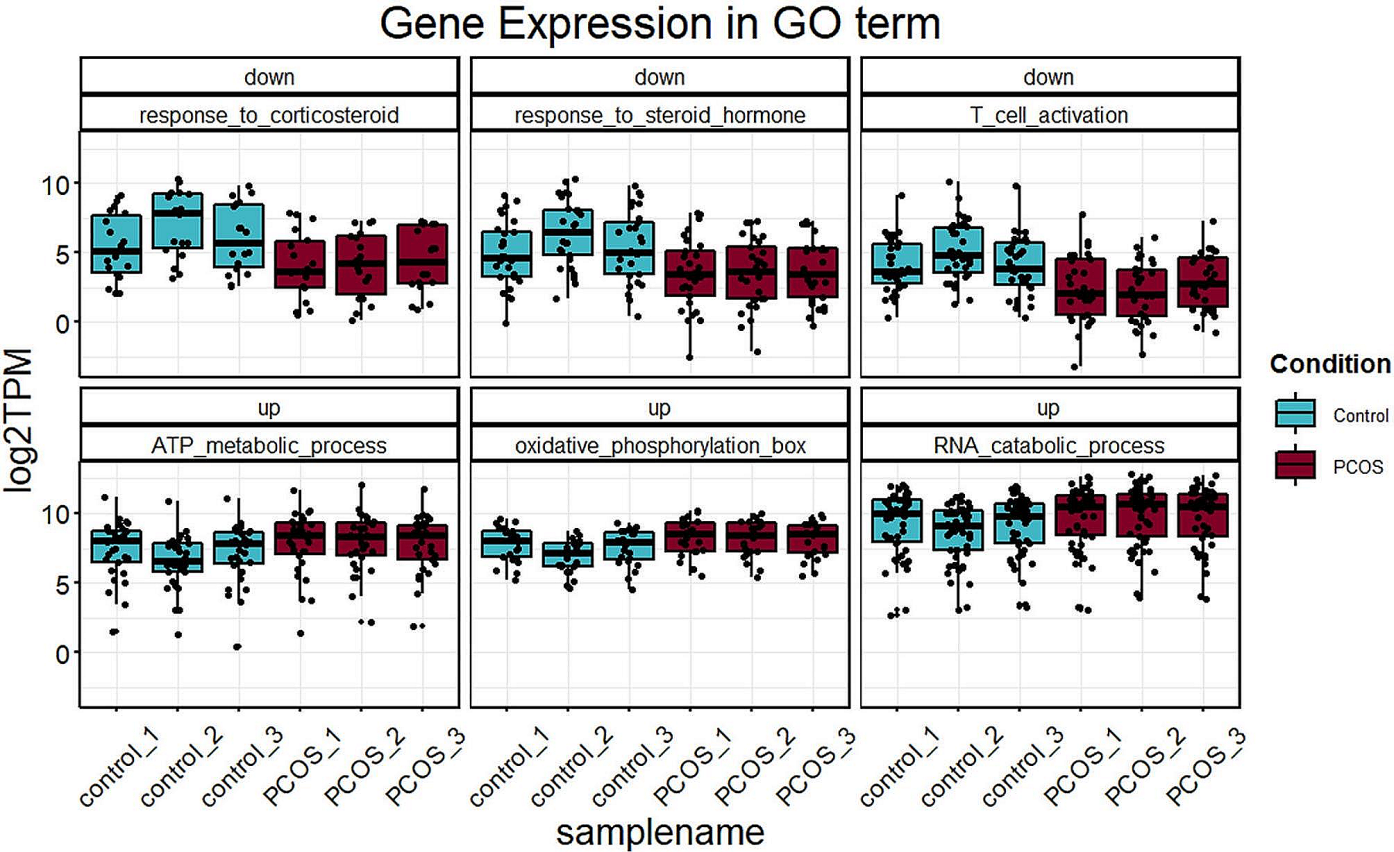
The gene expression of six key Go terms. The top row represents the down GO terms, and the the lower row represents the up- GO terms

**Fig. 6:**
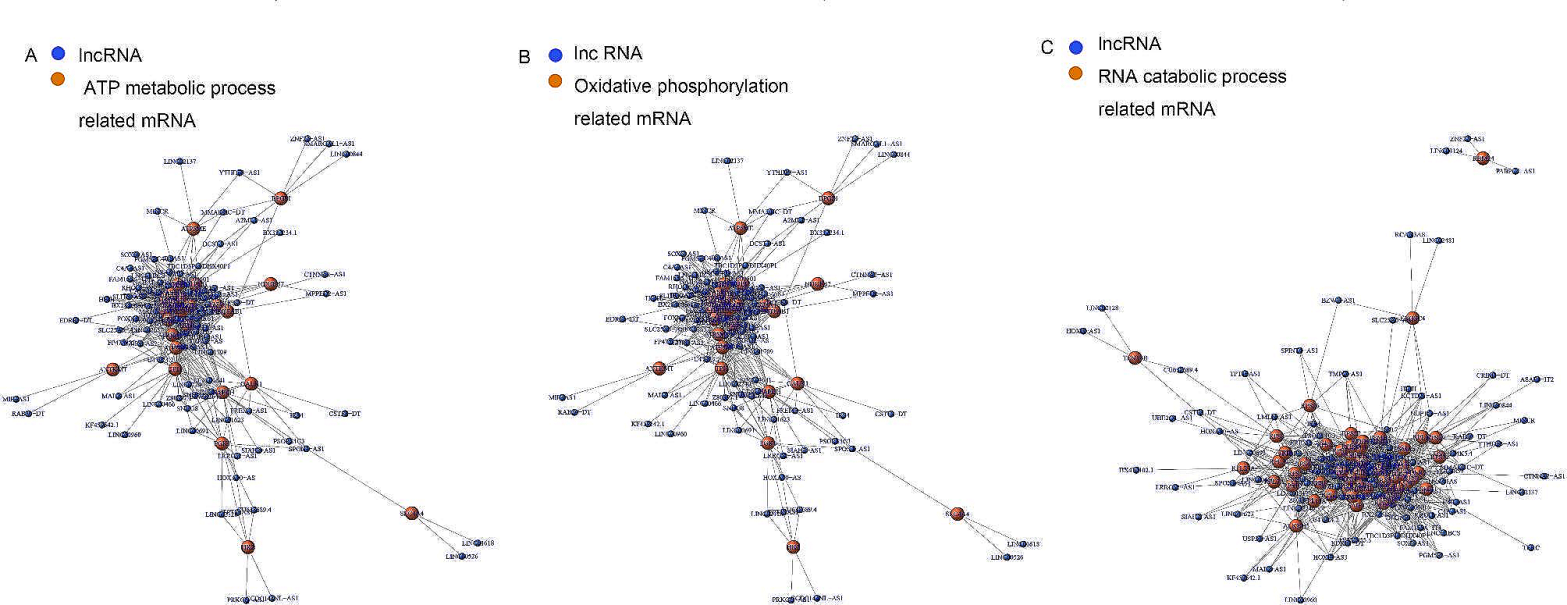
Analysis of lncRNA-mRNA co-expression profiles in pre-receptive endometrium of patients with PCOS. (A-C) The up-regulation of GO classification. Blue plots represent lncRNA, and orange plots represent ATP metabolic process related mRNA, oxidative phosphorylation related mRNA and RNA catabolic process related mRNA, respectively

**Fig. 7:**
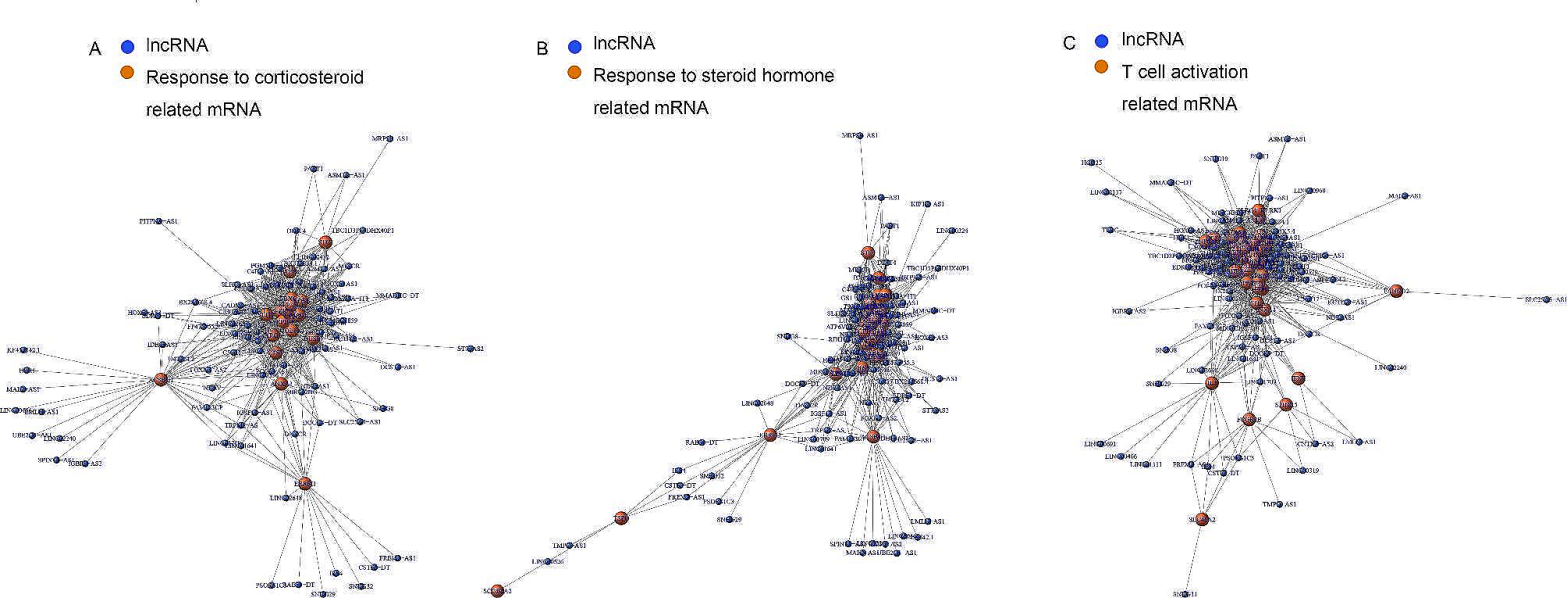
Analysis of lncRNA-mRNA co-expression profiles in pre-receptive endometrium of patients with PCOS. (A-C) The down-regulation of GO classification. Blue plots represent lncRNA, and orange plots represent response to corticosteroid related mRNA, response to steroid hormone related mRNA and T cell activation related mRNA, respectively

In order to identify the associated lncRNA-mRNA network and lncRNA biomarkers for predicting endometrial receptivity, we investigated the gene expression of six key Go terms (Fig. 5). We also further studied the differential lncRNA-mRNA co-expression network to reveal the potential regulatory roles of lncRNAs in endometrium of PCOS patients. Base on the up-regulation of GO classification, we analyzed the co-expression module of ATP metabolic process related mRNA and differentially expressed lncRNAs, the co-expression module of oxidative phosphorylation related mRNA and differentially expressed lncRNAs, the co-expression module of RNA catabolic process related mRNA and differentially expressed lncRNAs (Fig. 6A-C). Base on the down-regulation of GO classification, we analyzed the co-expression module of the co-expression module of response to corticosteroid related mRNA and differentially expressed lncRNAs, the co-expression module of response to steroid hormone related mRNA and differentially expressed lncRNAs, the co-expression module of T cell activation related mRNA and differentially expressed lncRNAs (Fig. 7A-C).

### Identification and validation of differentially expressed mRNAs and lncRNAs in pre-receptive endometrium of PCOS patients

**Fig. 8:**
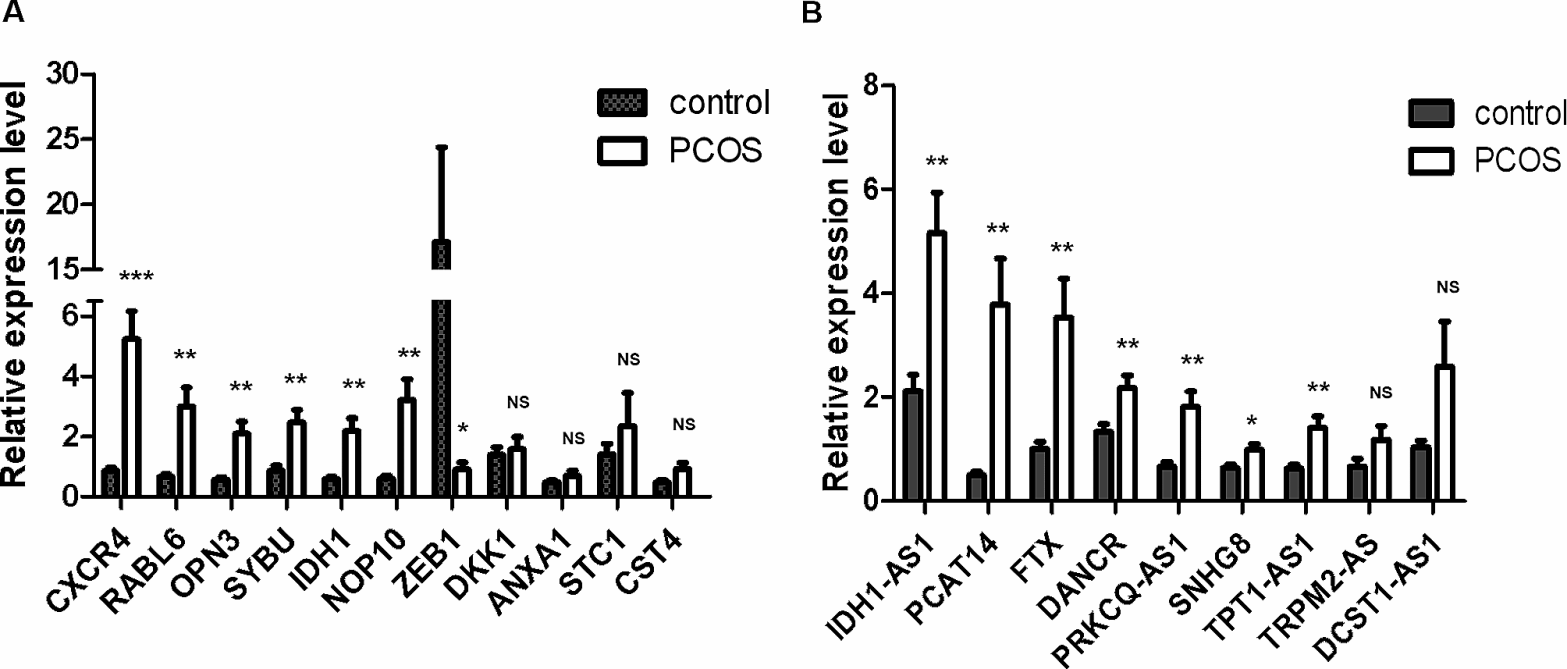
Identification and validation of differentially expressed mRNAs and lncRNAs in pre-receptive endometrium of PCOS patients. **(A)** The 11 key mRNAs were assayed by qRT-PCR on a sample set of 22 PCOS patients and 18 controls. **(B)** The 9 key lncRNAs were assayed by qRT-PCR on a sample set of 22 PCOS patients and 18 controls. *P < 0.05. **P < 0.01. ***P < 0.001 versus control group. NS: not significant, *P* > 0.05

The identified candidate mRNAs were assayed by qRT-PCR on a sample set of 40 patients (22 PCOS patients and 18 ovulation patients). As shown in Fig. 8A, the expression levels of 6 mRNAs CXCR4, RABL6, OPN3, SYBU, IDH1, NOP10 were significantly higher on 3 days after oocytes retrival in endometrium of PCOS than those in controls. The expression of ZEB1 was significantly decreased in endometrium of PCOS patients. The expression of DKK1, ANXA1, STC1 and CST4 was not statistically significant. qRT-PCR was performed to detect the expression of 9 lncRNAs, and validated that the expression of these 7 lncRNAs IDH1-AS1, PCAT14, FTX, DANCR, PRKCQ-AS1, SNHG8, TPT1-AS1 were significantly elevated in endometrium of PCOS patients. These results suggest that lncRNAs IDH1-AS1, PCAT14, FTX, DANCR, PRKCQ-AS1, SNHG8 and TPT1-AS1 may play a vital role in embryo pre-implantation. The expression of TRPM2-AS and DCST1-AS1 was not statistically significant (Fig. 8B).

In conclusion, the changes in the expression of lncRNAs (IDH1-AS1, PCAT14, FTX, DANCR, PRKCQ-AS1, SNHG8, TPT1-AS1)and mRNAs (CXCR4, RABL6, OPN3, SYBU, IDH1, NOP10) may possibly affect endometrial function in patients with PCOS before the implantation window, probably resulting in implantation failure of the embryo.

## Discussion

The typical pathophysiological change of PCOS, chronic anovulation, may impact endometrial function and morphology, leading to implantation failure, miscarriage, endometrial hyperplasia and even carcinoma [21]. However, the underlying mechanisms have not yet been thoroughly explored. In this study, we determined differentially expressed lncRNAs and mRNAs in pre-receptive endometrium between PCOS and control women by RNA-seq. Our study was the first to examine the pathophysiology changes of endometirum in PCOS using genome-wide expression profiles on 3 days after oocyte retrieval, which is the time of cleavage embryo transfer. We uncovered that 687 up-regulated mRNAs and 680 down-regulated mRNAs, 345 up-regulated lncRNAs and 63 down-regulated lncRNAs were significant differential expression in the PCOS patients, of which 9 lncRNAs and 11 mRNAs were validated by qRT-PCR. The expressions of 6 mRNAs (CXCR4, RABL6, OPN3, SYBU, IDH1, and NOP10) were significantly elevated in endometrium of PCOS patients. The expressions of 7 lncRNAs (IDH1-AS1, PCAT14, FTX, DANCR, PRKCQ-AS1, SNHG8, and TPT1-AS1) were significantly elevated in endometrium of PCOS patients. These differentially expressed lncRNAs and mRNAs may regulate endometrial receptivity and predicted their functions.

This study identified lncRNA biomarkers with predictive value of endometrial receptivity and found out the related lncRNA-mRNA network. The differential lncRNA-mRNA co-expression network revealed the potential regulatory roles of lncRNAs in endometrium of PCOS patients. The differentially expressed lncRNAs related to six key Go terms of mRNA were mainly involved in ATP metabolic process, oxidative phosphorylation, RNA catabolic process and response to corticosteroid, as well as response to steroid hormone and T cell activation. The altered lncRNA-mRNA network in endometrium of PCOS supported the hypothesis that impaired endometrial receptivity may play a critical role in implantation and pregnancy failure among PCOS patients.

A group of key mRNAs associated with altered abundance in the endometrium of PCOS women were related to ATP metabolic process. We also analyzed the co-expression module of ATP metabolic process related mRNA and differentially expressed lncRNAs. Energy balance is critical for the normal function of reproductive system. The metabolic abnormalities may contribute to the development of subfertility in PCOS. The prevalence of metabolic syndrome (MetS) in PCOS was 28.8% [22]. Altered fertility was commonly linked to metabolic dysfunctions including dyslipidemia and insulin resistance among PCOS women [23]. Abnormalities in pancreatic ATP synthase β subunit was found in mice with PCOS and type II diabetes comorbidity [24]. Besides, ATP inhibits human endometrial stem cell’s migration and proliferation [25] and mediates blastocyst-endometrium crosstalk in human [26]. These studies suggest that ATP metabolic processes may affect endometrial function in PCOS, which is consistent with our study. Some studies indicate that ATP release from luminal epithelial cells stimulates decidualization in mice via the P2Y2 receptor [27]. However, our study did not further investigate the specific mechanism. There were studies showed that resveratrol may improve endometrial function by improving ATP metabolism, restoring glycotic process and rehabilitating the number of granular cells [28]. Therefore, some drugs such as resveratrol may improve endometrial function through the process of ATP metabolism.

PCOS is associated with insulin resistance (IR) and altered muscle mitochondrial oxidative phosphorylation. Significant transcriptomic changes occurred in primary human endometrial stromal cells in a stepwise fashion. After embryo invasion, oxidative phosphorylation, mitochondrial organisation, and p53 signalling pathways were significantly altered in primary human endometrial stromal cells [29]. In our study a large proportion of differentially expressed mRNAs were involved in oxidative phosphorylation. Activation of oxidative phosphorylation in TP53-inactive endometrial carcinomas with a poor prognosis [30]. Maria M Szwarc revealed that SRC-2 knockdown reduces endometrial cancer cell proliferation and anchorage-independence [31]. SRC-2 can maintain cellular glycolytic capacity and oxidative phosphorylation, a process necessary for endometrial cancer cell proliferation. Both the metabolic and oxidative phosphorylation pathways are dysregulated in PCOS patients’ oocytes and cumulus cells [32]. Therefore, oxidative phosphorylation may be involved in the regulation of endometrial function.

The alteration of genes related to the RNA catabolic process in the endometrium of PCOS was showed in this study, and K H Sadek indicated that housekeeping genes were variable in stability in endometrium of healthy and PCOS women [33]. An increase in transcriptional regulation and mRNA stability was observed in PCOS patients with cholesterol side-chain cleavage gene expression [34]. Yuhua Z Farnell also indicated that the interaction between estradiol and selective modulators of its receptor could affect mRNA transcription and stability in human endometrial adenocarcinoma cells [35]. Therefore, altered expression of RNA catabolic process in endometrium from patients with PCOS may be related to the function of endometrium.

T cells play a crucial role in implantation and maintenance of pregnancy. The immunomodulatory mechanisms at the foetus-maternal interface remain unclear for this complex system. Understanding the role of T cells is a key element in understanding the endometrial immune system. T cells, the dominant lymphocytes in endometrium, could populate the decidua, but their precise function is unclear. Decidual CD4^+^ T (dCD4^+^ T) cells are essential for the induction and maintenance of maternal immune tolerance [36]. Decidual T cells may contribute to the maintenance of pregnancy by producing LIF, interleukin (IL)-4, IL-10 and M-CSF. T cells, which can produce LIF and IL-4, are also found in cumulus oophorus [37]. The activation and cytokine secretion levels of T cells in omental fat tissue and peripheral blood are linked to testosterone level and insulin resistance among PCOS women [38]. These findings suggest that in the pathogenesis of PCOS, metabolic changes in both CD4^+^ and CD8^+^ T cells that favour Th2 differentiation and glycolysis are important [39], and peripheral blood inflammatory-immune cells may serve as potential predictors of infertility in PCOS patients [40].

Aberrant endometrial receptivity in PCOS may result from altered expression of genes involved in steroid hormone synthesis. Corticosteroid and steroid hormone may affect the oocyte, the future embryo, and the endometrium. Expression profiling experiments using endometrium of PCOS patients in our study also provided evidence that corticosteroids and steroid hormones are important in the pathogenesis of PCOS endometrium. Hormones involved in human bone metabolism, including sex hormones, growth hormone and parathyroid hormone, show a disturbed pattern of production and secretion in PCOS [41]. Given the LH receptor expression in the endometrium [42, 43], high LH level in PCOS could directly influence endometrial pathophysiology. In high responders, decreased embryo implantation has been reported regardless of embryo quality, possibly due to a negative effect of supraphysiological circulating estradiol on the endometrium [44, 45]. Driven by steroid hormones, the cyclic changes of human endometrium during normal menstrual cycle are orchestrated by a plethora of epigenetically-regulated genes [46]. Corticosteroid signalling dysfunction in decidual cells has been linked to increased natural killer cell density in human endometrium during the periimplantation window [47].This study explored differences expression of corticosteroid and steroid hormone related genes between ovulation and PCOS women. Corticosteroid and steroid hormone profiling were different between ovulation and PCOS women.

Our study has the following limitations: [1] The heterogenic demographic and baseline characteristics of the two groups may introduce bias to finding interpretation. However, strict spatio-temporal restrictions on tissue sampling had been set to minimize other confounders; [2] We had a small sample size, but external validation among both PCOS and normal ovulatory patients were performed to verify the reliability of our findings; [3] We did not analyze the pregnancy outcomes of the included patients, since they were asked to use contraception for at least 1 month after endometrial biopsy. Future studies on pregnancy outcome are warranted for adequate interpretation of the GE changes we found.

The strength of our study was that, for the first time, we detected the pathophysiology changes in the endometirum of PCOS patients using genome-wide expression profiles at 3 days after oocyte retrieval, which is the time of cleavage embryo transfer. This study also identified lncRNA biomarkers with predictive value of endometrial receptivity and found out the related lncRNA-mRNA network. The differential lncRNA-mRNA co-expression network revealed the potential regulatory roles of lncRNAs in endometrium of PCOS patients, which provided a basis for the mechanism of endometrial receptivity in patients with PCOS, and partially served an explanation why PCOS women have an impaired reproductive outcome in clinic. This will provide the possibility to improve the pregnancy rate in patients with PCOS. Future studies should investigate the underlying mechanism, and lncRNAs may be developed as new potential diagnostic and therapeutic targets, which has important clinical implications.

## Conclusion

Our results determined the characteristics and expression profile of endometrial in PCOS patients in pre-receptive phase. The possible pathways and related proteins of endometrial receptivity disorders were found, and lncRNAs may be developed as a predictive biomarker of endometrial receptivity.

## Data Availability

The datasets supporting the conclusions of this article are available in. Gene Expression Omnibus GSE226146. https://www.ncbi.nlm.nih.gov/geo/query/acc.cgi?acc=GSE226146.
